# Preventing and Managing Chronic Conditions In Older Adults: Lessons Learned From the NHATS and NSOC 2017

**DOI:** 10.1093/geroni/igaa057.2873

**Published:** 2020-12-16

**Authors:** Rose Ann DiMaria-Ghalili, Cheryl Monturo

## Abstract

According to the Centers for Disease Control, 85% of older adults have at least one chronic health condition, and 60% percent have at least two chronic conditions. This symposium explores strategies to prevent and manage chronic conditions (nutritional status, medication management, wound care, and physical function) using data from the 2017 National Health and Aging Trends Study (NHATS) and corresponding National Study on Caregiving (NSOC). The datasets include a nationally representative sample of US older adults (NHATS) and their caregivers (NSOC). In addition to survey questions, the 2017 NHATS cohort submitted dried blood samples which include inflammatory biomarkers (hs-C reactive protein [hsCRP] and interleukin-6 [IL-6]). All individual presentations report on weighted data from the analysis to more accurately reflect the US population. In the first paper, DiMaria-Ghalili explores the prevalence of and factors related to malnutrition in community-dwelling and residential living older adults. In the second paper Coates examines the extent to which source of purchased medications impacts the occurrence of self-reported medication mistakes and hospitalizations in community-dwelling participants who managed medications independently. In the third paper, Hathaway compares the socio-demographic, nutrition, and inflammatory profile of older adults with and without wounds. In the fourth paper, Sefcik examines the relationship between the frequency of community-dwelling older adults going outside and physical function. Collectively, findings provide insight into the experiences of vulnerable older adults with chronic conditions informed from the NHATS and NSOC datasets. The symposium will conclude with a discussion by Monturo on implications for research, policy and practice.

